# Restaurant outbreak of Legionnaires' disease associated with a decorative fountain: an environmental and case-control study

**DOI:** 10.1186/1471-2334-7-93

**Published:** 2007-08-09

**Authors:** Rosalyn E O'Loughlin, Lon Kightlinger, Matthew C Werpy, Ellen Brown, Valerie Stevens, Clark Hepper, Tim Keane, Robert F Benson, Barry S Fields, Matthew R Moore

**Affiliations:** 1 Respiratory Diseases Branch, Division of Bacterial Diseases, National Center for Immunization and Respiratory Diseases, Centers for Disease Control and Prevention, Mailstop C-23, 1600 Clifton Road NE, Atlanta GA 30333, USA; 2 Epidemic Intelligence Service Program, Office of Workforce and Career Development, Centers for Disease Control and Prevention, 1600 Clifton Road NE, Atlanta GA 30333, USA; 3 State of South Dakota Department of Health, 600 East Capitol Avenue, Pierre, SD 57501, USA; 4 Legionella Risk Management, Inc, 31 Marian Circle, Chalfont, PA 18914, USA

## Abstract

**Background:**

From June to November 2005, 18 cases of community-acquired Legionnaires' disease (LD) were reported in Rapid City South Dakota. We conducted epidemiologic and environmental investigations to identify the source of the outbreak.

**Methods:**

We conducted a case-control study that included the first 13 cases and 52 controls randomly selected from emergency department records and matched on underlying illness. We collected information about activities of case-patients and controls during the 14 days before symptom onset. Environmental samples (n = 291) were cultured for *Legionella*. Clinical and environmental isolates were compared using monoclonal antibody subtyping and sequence based typing (SBT).

**Results:**

Case-patients were significantly more likely than controls to have passed through several city areas that contained or were adjacent to areas with cooling towers positive for *Legionella*. Six of 11 case-patients (matched odds ratio (mOR) 32.7, 95% CI 4.7-∞) reported eating in Restaurant A versus 0 controls. *Legionella pneumophila *serogroup 1 was isolated from four clinical specimens: 3 were Benidorm type strains and 1 was a Denver type strain. *Legionella *were identified from several environmental sites including 24 (56%) of 43 cooling towers tested, but only one site, a small decorative fountain in Restaurant A, contained Benidorm, the outbreak strain. Clinical and environmental Benidorm isolates had identical SBT patterns.

**Conclusion:**

This is the first time that small fountain without obvious aerosol-generating capability has been implicated as the source of a LD outbreak. Removal of the fountain halted transmission.

## Background

On average, 1675 cases of legionellosis are reported to the Centers for Disease Control and Prevention (CDC) annually by state health departments, the majority of which are Legionnaires' disease (LD) (CDC, unpublished data). It is estimated that between 8,000 and 18,000 persons are hospitalized with LD in the United States each year, suggesting that under-diagnosis or under-reporting are common [1]. One species of *Legionella, Legionella pneumophila*, is responsible for 90% of reported cases of LD in the United States and the majority of these are caused by *Legionella pneumophila *serogroup 1 (*Lp*1) [2,3].

Eighty percent of cases of LD are sporadic [4] and the source of *Legionella *for these cases is rarely known. In contrast, outbreaks, while rare, provide an opportunity to identify common sources of *Legionella*. Community LD outbreaks have frequently been associated with cooling towers [5-13], and whirlpool spas [14-17], and less frequently with evaporative condensers [18], air-scrubbers [19], supermarket misters [20], potable water [21,22], and decorative fountains [23].

In a three week period in June and July 2005, seven cases of LD among residents of Rapid City were reported to the South Dakota Department of Health (SDDH). In the previous 10 years, only two confirmed cases of LD had been reported to the SDDH from Rapid City. (Personal communication, Dr. Kightlinger) No common exposures among case-patients were initially apparent. In July 2005, SDDH and CDC began an epidemiological and environmental investigation to identify the source of the outbreak and to prevent additional cases.

## Methods

### Epidemiologic investigation

We defined a case as a resident of, or visitor to, Rapid City, South Dakota who was diagnosed by a physician, either clinically or radiographically, with community-acquired pneumonia (CAP) with onset after May 1, 2005 and who had laboratory confirmation of LD by culture of *Legionella*, by urinary antigen test for *Lp*1, by a four-fold or greater rise in serum antibody titer to *Lp*1, or detection of specific *Legionella *antigen by direct fluorescent antibody staining.

To enhance surveillance for additional cases of LD associated with Rapid City, alerts regarding the increase in cases were made locally (July 2005), statewide (July 2005), and nationwide (August 2005), to public health practitioners, physicians and health facilities. Clinicians were encouraged to perform urine testing for LD for all hospitalized patients with CAP and to report any cases to SDDH. To determine whether the increase in cases represented an outbreak or simply increased diagnostic testing, we examined trends in admissions for CAP to Rapid City Regional Hospital, and *Legionella *urine antigen testing. Hypothesis generating interviews were held with the first six case-patients or their surrogates in an attempt to identify any common exposures.

We conducted a matched case-control study that included the first 13 of the 18 case-patients to identify exposures associated with LD. The remaining five case-patients were not enrolled as they were reported after the source of the outbreak had been identified and publicized. Four controls per case (n = 52) were matched on underlying illness category (healthy, underlying illness or immunocompromised) and smoking status. As all enrolled case-patients were white, older than 50 years, and residents of Rapid City, we restricted our controls to white persons over the age of 50 years from Rapid City. Controls were selected randomly from among persons attending the local hospital's emergency department within seven days before or after the illness onset date of the corresponding case-patient. Potential controls who had a diagnosis of CAP in the last six months, who were residents of a long-term care facility, or who had been away from home for five or more nights during the two weeks of interest were excluded. We interviewed all case-patients or their surrogates in person and interviewed controls either in person, or by phone if they were unwilling to meet an investigator in person.

During interviews with case-patients and controls, we used a map of Rapid City and a calendar to prompt recall of activities during each two-week period of interest. Case-patients and controls were asked to refer to their diaries, check-books, and receipts to further assist with recall. Questions were general, such as "did you visit any restaurants?" or "did you visit any supermarkets?" If the answer was in the affirmative then the person was asked to specify which ones were visited. Reported routes of travel throughout the city were traced on a map which was divided into 180 grids, where each grid represented 1.29 km^2^, and the grid coordinates were recorded.

Matched analysis of the matched sets using exact methods in SAS version 9.1 (Cary, NC) was used to compute matched odds ratios (mOR) and 95% confidence intervals (CI).

### Environmental investigation

We conducted an environmental assessment of the city to locate aerosol-generating devices. The devices were identified by aerial, driving, and walking surveys of the city and through contacts with city managers, local property managers, water treatment companies, and industrial plumbing contractors. In September 2005, SDDH contracted an engineering consultant (TK) to assist in the environmental investigation and remediation efforts. Sample collection and analysis were performed according to standard procedures [24]. Between July and November 2005, 291 environmental samples (201 water, 90 biofilm swabs) were collected from 123 potential sources at 73 sites throughout Rapid City. Sampling continued through the course of the outbreak initially concentrated in the documented areas of exposure of case-patients. Some sites were sampled more than once. Potential sources sampled included cooling towers, chiller units, supermarket misters, swamp coolers, decorative fountains, whirlpool spas, the municipal water system, wells and a stream. We also sampled the potable water system from the first seven case-patient homes, but as we found only one sample positive for *Legionella*, we discontinued this sampling as we concluded that acute dissemination of *Legionella *via the municipal water system was an unlikely explanation for this outbreak.

A one-liter water sample and, where appropriate, a biofilm swab were taken from each location. Water samples were collected in sterile plastic bottles containing a 0.1% sodium thiosulfate solution. Samples were shipped to the *Legionella *Laboratory at CDC (Atlanta, GA) or to the Special Pathogens Laboratory, Pittsburgh Veterans Administration Medical Center where *Legionella *was identified using previously described methods [3]. Clinical and environmental isolates were compared using monoclonal antibody (MAb) subtyping [25] and sequence based typing (SBT) [26].

Research was conducted in compliance with guidelines of the U.S. Department of Health and Human Services as they apply to epidemic investigations by the U.S. Public Health Service, CDC.

## Results

### Epidemiologic results

**Figure 1:**
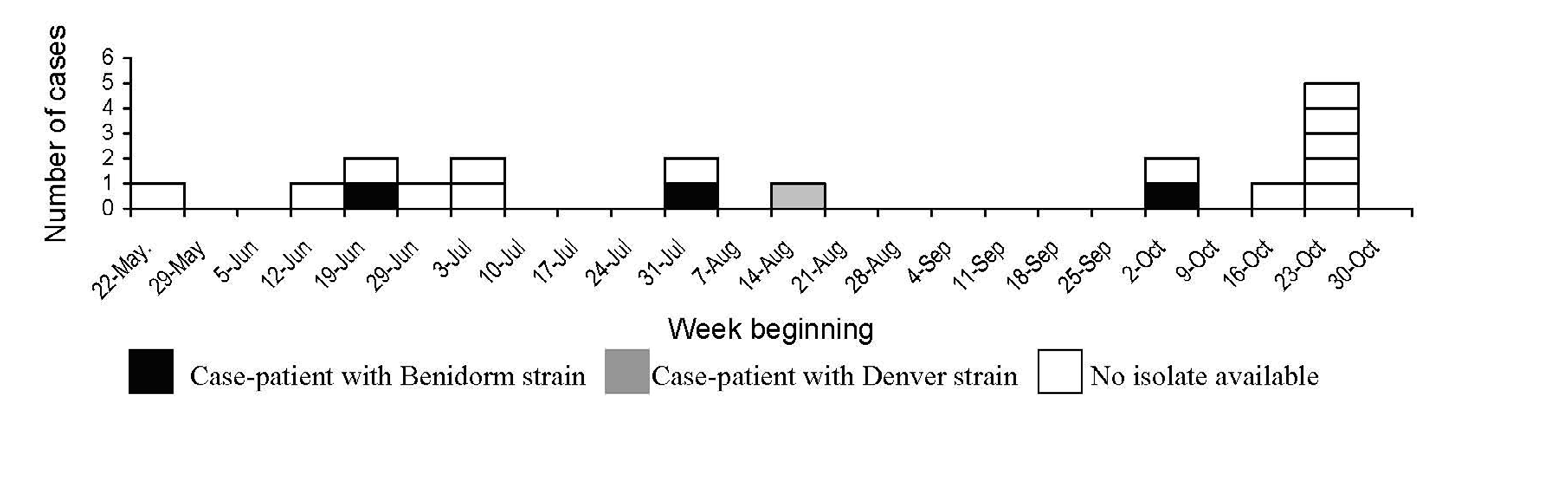
Epidemiological curve of case onsets of LD in Rapid City, 2005.

Between June 27 and July 18, 2005, SDDH was notified of seven cases of LD among residents of Rapid City. Enhanced surveillance detected an additional 11 case-patients for a total of 18 cases being reported by November 2005. Sixteen case-patients were residents of Rapid City and two were South Dakota residents who had visited Rapid City. Fourteen (77.8%) of 18 patients were hospitalized, and 1 (5.6%) died. Onset dates ranged over a five-month period from May 26, 2005 to October 29, 2005 (Figure 1). Ten patients (56%) were men. Ages ranged from 51 to 88 years (median, 67 years). Eleven (61.1%) patients had at least one medical risk factor for LD; diabetes mellitus was the most common (n = 7). One patient was a current smoker and seven were former smokers. The diagnosis of LD was made for all patients by detection of Lp1 antigens in urine by enzyme immunoassay (EIA). Four case-patients were also diagnosed by isolation of *Lp1 *from respiratory secretions; 3 were Benidorm strain (MAb pattern 1, 2, 5, 7), a common outbreak strain, and one was Denver strain (MAb pattern 1, 3, 6), a relatively rare strain not previously associated with outbreaks (Figure 1). Data from Rapid City Regional Hospital showed an increase in *Legionella *urine antigen tests from November 2004 with no unusual increase in CAP or LD.

Thirteen of the 18 case-patients and 52 matched controls were enrolled in the case-control study. The median age of case-patients was 67 years (range 51–86) compared to 61 years (range 50–92) for controls. All case-patients and controls were residents of Rapid City and none were current smokers. Seven (53.9%) of the 13 case-patients enrolled in the case-control study had a chronic underlying condition or were immunosuppressed.

**Figure 2:**
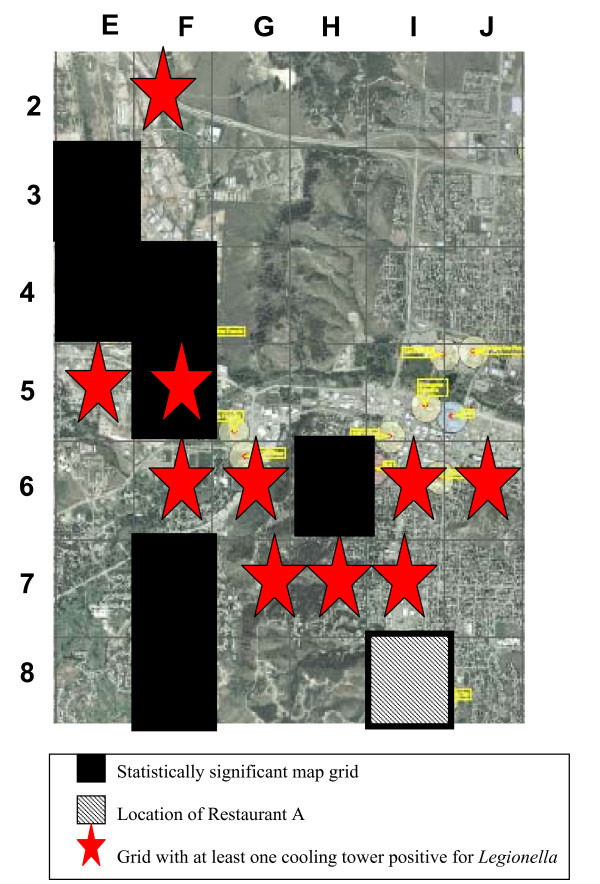
Segment of map of Rapid City.

Case-patients were significantly more likely than controls to have passed through seven map grids (Table 1). Some of these grids were in, or adjacent to, grids with cooling towers colonized with *Legionella *(Figure 2). This initially supported the hypothesis that a cooling tower was the source of the outbreak. This was strengthened by the fact that no two patients lived or worked in the same building and among the first six case-patients, we could not place more than two case-patients in any one building or adjacent city blocks. Case-patients were not more likely than controls to have spent time outdoors (Table 1).

**Table 1:** Matched odds ratio for selected exposures

Activity	Cases (%) N = 13*	Controls (%) N = 52*	Matched OR	95%CI
Passed through grid E3	10/13 (77)	12/52 (21)	10.3	2.1–100.9
Passed through grid E4	9/13 (69)	15/52 (29)	6.8	1.3–68.5
Passed through grid F4	9/13 (69)	12/52 (23)	9.1	1.7–90.9
Passed through grid F5	9/13 (69)	15/52 (29)	12.1	1.5–558.5
Passed through grid F7	11/13 (85)	23/52 (44)	6.5	1.3–65.4
Passed through grid F8	10/13 (77)	18/52 (35)	4.9	1.2–29.3
Passed through grid H6	12/13 (92)	31/52 (60)	12.0	1.3–597.4
>1.5 hours outdoors weekdays	9/13 (69)	30/52 (57)	1.6	0.4–8.0
>1.5 hours outdoors weekends	6/13 (46)	30/52 (57)	0.65	0.2–2.5
Visited any restaurant	13/13 (100%)	29/52 (56)	17.7	2.5–8
Reported eating in Restaurant A on first contact with SDDH	6/11 (55)	0/44 (0)	32.7	4.7–8
Visited any food store	7/13 (54)	38/52 (73)	0.5	0.1–1.9
Visited any medical center	7/13 (54)	43/52 (83)	0.2	0.03–1.1
Traveled on main city thoroughfare	10/12 (83)	33/48 (69)	2.2	0.4–22.0

*Denominators vary as not all case-patients answered all questions or were excluded because they were unsure.

Table 1 presents matched odds ratios (mOR) for selected exposures. Apart from passing through certain map grids, visiting a restaurant was the only other activity with a statistically significant odds ratio. Six of 11 case-patients versus 0 controls (mOR 32.7) reported eating at Restaurant A during their first interview (information was unavailable for one case-patient and one case-patient was unsure). Six of seven case-patients were interviewed by the end of July and two reported eating at Restaurant A. Nine of 10 case-patients were interviewed by the end of July and three reported eating at Restaurant A. Finally, 12 of 13 case-patients were interviewed by the end of October and six reported eating at Restaurant A (the mOR is presented for the 6 of 11 cases who answered the restaurant question). After the public disclosure of Restaurant A as the source of the outbreak on October 29, 2005, all case-patients were re-contacted by SDDH to verify their exposure status and exposure date, and to ask how long they had spent in the waiting area where the fountain was located. Controls were not re-contacted. Simultaneously, some case-patients were prompted to contact SDDH to say they had eaten at Restaurant A despite not mentioning this in their initial interview. Ultimately, 10 (83%) of 13 case-patients reported eating in Restaurant A within 14 days of their illness onset (two case-patient reported that they ate at Restaurant A but were unsure of when, and one case-patient was not reported to have eaten in Restaurant A). Of the five case-patients not enrolled in the case-control study because they were reported after the fountain had been removed, all reported eating in Restaurant A. Restaurant A was located in map grid I8 which had a non-significant odds ratio (Figure 2).

### Environmental results

*Legionella *was isolated from 43 (35.0%) of 123 potential environmental sources and from 95 of 291 (32.6%) samples (Table 2). *Legionella pneumophila *was the most common species identified and *Lp1 *was the predominant serogroup with 25 (58.1%) of 43 sources positive for *Lp1*. Among the *Lp1 *isolates that were typed, strains reacting with MAb 1 were most common, suggesting the presence of type strain Camperdown (MAb 1) and/or Oxford 4032E (MAb 1, 6). Due to a shortage of MAb 6 we were unable to distinguish between Camperdown and Oxford for all isolates although the few that were tested were Oxford.

**Table 2:** Environmental sources positive for *Legionella *bacteria

	**Sources**		**Samples**	
**Potential environmental sources**	**Number investigated**	**Number (%) positive**	**Number collected**	**Number (%) positive**

Case-patient homes	7	1 (14.3)	37	1 (2.7)
Cooling towers	43	24 (55.8)	116	68 (58.6)
Chillers	6	5 (83.3)	10	7 (70)
Swamp coolers and sumps	9	5 (55.6)	12	5 (41.7)
Decorative fountains and ornamental waterfalls	22	2 (9.1)	42	2 (4.8)
Municipal, local and industrial water sources	22	3 (13.6)	39	6 (15.4)
Supermarket misters	8	2 (25.0)	20	2 (10)
Whirlpool spa	1	0 (0.0)	2	0 (0.0)
Restaurant A				
Fountain	1	1 (100)	4	4 (100)
Other water sources	4	0 (0.0)	9	0 (0.0)
Total	123	43 (34.7)	291	95 (32.6)

The outbreak strain, Benidorm (MAb pattern 1, 2, 5, 7), was isolated from only one source, a decorative fountain in Restaurant A. Swabs were taken from the fountain basin, nozzle, and internal lamp and a water sample was taken. All were positive for Benidorm strain. We compared all Benidorm clinical and environmental isolates and found identical SBT patterns for the genes *fla*A, *pil*E, *asd*, *mip*, *momp*S, *pro*A, i.e., the pattern 4,7,11,3,11,12, respectively. The decorative fountain had a water *Legionella *colony count of 3000 cfu/ml. Of the remaining water sources positive for *Legionella *that had colony counts recorded, 10 had counts less than 50 cfu/ml, 10 had counts between 50 and 499 cfu/ml, eight had counts between 500 and 1499 cfu/ml, and three had counts above 1500 cfu/ml.

**Figure 3:**
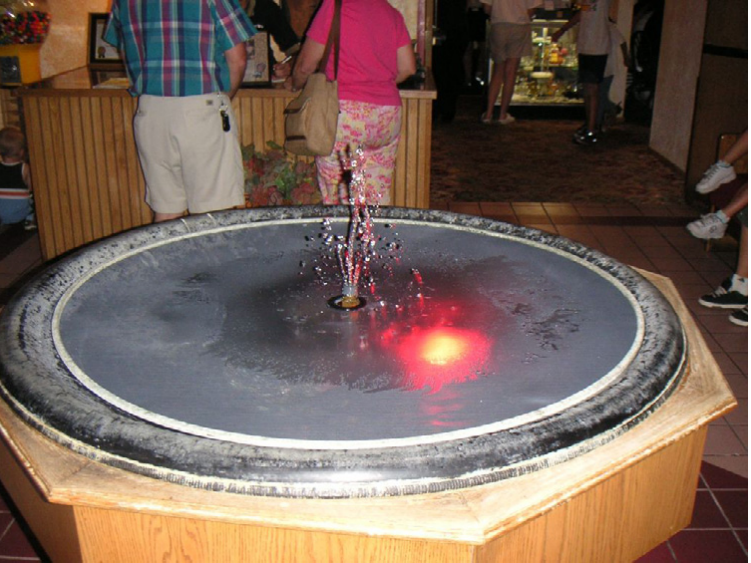
Decorative fountain situated in the lobby of Restaurant A.

The decorative fountain located in the lobby of Restaurant A was sampled on October 24, 2005, turned off at that time of sampling, and removed a few days later. The diameter of the basin of the fountain was approximately 1.2 m with a central water column of approximately 30 cm. The basin was covered with mesh and three colored lights were submerged in the water (Figure 3). The fountain operated for 10–12 hours per day. Restaurant A had seating capacity for 284 people. Case-patients reported a median of four minutes (range 0.5–17.5 minutes) spent in the lobby where the fountain was located. No employees of Restaurant A were reported ill. No cases of LD with an onset date of more than five days after the date the fountain ceased operation were reported in Rapid City. Denver strain, which was identified from one clinical isolate, was not identified from any environmental samples.

## Discussion

We believe that the source of this outbreak was the decorative fountain in Restaurant A for a number of reasons. The epidemiological evidence showed that even with poor case-patient recall the odds ratio from the case-control study showed a strong association between eating at restaurant A and having LD. The environmental investigation found Benidorm strain in the fountain in Restaurant A but not in any other potential environmental source. In addition, the strain isolated from the fountain and the three patient isolates were identical by SBT analysis. The colony count observed in the fountain, while very high, should be viewed as supportive but not decisive evidence of the fountain being the source.

This outbreak was unusual in that it lasted several months, a source that on observation appeared to generate only a small aerosol was implicated, and the case-patients had very short exposure periods. Previously a decorative fountain located in a hotel lobby was associated with an outbreak of LD [23] and a decorative fountain in a restaurant was linked with an outbreak of Pontiac Fever [27]. In addition, a decorative fountain located outdoors was the suspected source of an outbreak of LD in Portugal [28]. These fountains were significantly larger than the fountain implicated in this outbreak and were likely to have generated a larger aerosol. The fountain in Restaurant A was one of the many potential sources investigated early in the outbreak. Only sources that had a reasonable epidemiological link and that were considered to be a plausible source were sampled. Our initial assessment of the fountain in Restaurant A in July led us to conclude that it was an unlikely source because it appeared to generate very little aerosol, and because at that point only two case-patients reported having eaten at Restaurant A. Therefore, it was not sampled at that time but in October when we found a stronger epidemiological link. The fountain was reported to be out of operation for approximately 30 consecutive days during the time of this outbreak. Although the exact dates were unavailable to us, they appeared to correspond with the period on the epidemic curve when there were no cases. Rather than remediate the fountain in Restaurant A the fountain was permanently removed.

Our initial hypothesis was that a cooling tower was the source of this outbreak for three reasons. Firstly, we could find no common exposures and could not initially place more than two case-patients in any one building, leading us to believe that the source was outdoors. Previous studies have shown infections occurring as far as 6 km from an external source indicating that the wind is capable of transporting *Legionella *plumes [5]. However, exposure to contaminated cooling tower drift can occur indoors but in this situation there was no geographical clustering of patient homes which might have been expected if infection was as a result of a drift. Secondly, we found several cooling towers with *Legionella *in the city close to areas where people shopped and worked, and to map grids through which case-patients were significantly more likely than controls to have traveled. Apart from exposure to cooling towers (using map grids as a proxy), analysis of potential exposures where more than half the case-patients had visited did not initially show any significant differences between case-patients and controls. This hypothesis was strengthened by the timing of the outbreak at the start of summer with many towers bearing heavier cooling loads and running in excess of 100% capacity due to a hotter than usual summer in South Dakota. Thirdly, as outlined in the background, cooling towers are common sources of community outbreaks of LD.

Clinicians at the local hospital in Rapid City had adopted a policy in November 2004 of more intensive diagnostic testing for patients with CAP so that antibiotic therapy could be targeted. *Legionella *urine antigen testing therefore increased prior to detection of this outbreak. This highlights the importance of appropriate diagnostic testing in patients with CAP. While many patients with LD can be treated successfully and empirically with antibiotics currently recommended for CAP, this outbreak shows that the use of etiology-specific diagnostic tests can lead to a public health intervention that prevents future cases of LD. Furthermore, increasing physician awareness and use of the urinary antigen testing, through our recommendation during the outbreak to test all new cases of CAP for LD, may have improved patient management and contributed to the relatively low case-fatality rate. The availability of clinical isolates of *Legionella *from 4 case-patients was critical in allowing us to identify the source of the outbreak. In the United States there has been a steady decline in the proportion of LD cases diagnosed by culture since the introduction of urine antigen testing [2]. However, as this outbreak demonstrates, diagnostic testing of persons with CAP should include collection of urine for antigen testing and respiratory specimens for culture of *Legionella *whenever possible. This recommendation is included in the recently updated Infectious Disease Society of America/American Thoracic Society (IDSA/ATS) guidelines on the management of CAP in adults [29].

*Legionella *was cultured from over half (55%) of the cooling towers tested, several of which were positive for *Lp1*. Although the outbreak was assumed to be caused by Benidorm strain, which was not found in any of the cooling towers, the presence of *Legionella *in so many sources, some with high colony counts, and with the potential to aerosolize is worrying. Other studies have shown that detectable levels of *Legionella *are present in many cooling towers and other building water systems [30,31]. Some researchers advocate measurement of colony counts as a predictor of disease risk [30]. However, because measurement of colony counts is not standardized, no other source was initially evident, and because there is no known safe level of *Legionella*, we applied the precautionary principle and recommended that all positive towers be remediated regardless of their colony count. SDDH employed a *Legionella *consultant (TK) to manage remediation of the cooling towers in accordance with published guidelines [32], and to hold cooling tower maintenance workshops for industry and businesses in Rapid City and two other South Dakota cities.

Our investigation had certain limitations. While it is understandable that individuals recovering from serious illnesses would have some difficulties with recall of activities 1 to 2 weeks earlier, poor recall delayed our ability to identify Restaurant A as a potential source of the outbreak. We could have asked our case-patients and controls whether they visited each individual shop, restaurant, etc. in Rapid City. However, this would have made our already lengthy questionnaire unwieldy. Case-patients were re-interviewed about their exposure to Restaurant A but controls were not re-interviewed. Therefore, we have not presented new matched odds ratios as they may be biased. Given the magnitude of the odds ratio (mOR 32.7) based on the initial case-patients' reported attendance at Restaurant A and given that the Benidorm strain was found only in the fountain in Restaurant A, we believe that not re-interviewing controls has not weakened our conclusions that the fountain in Restaurant A was the source of this outbreak. We excluded the last five cases from the case-control study because they were reported after the source of the outbreak was published and we were concerned that their responses may have been biased. We believe that these cases appeared rather suddenly because media attention may have led to increased medical care-seeking, increased diagnostic testing, increased reporting, or all three.

We were unable to explain the presence of Denver strain in the clinical isolate of one case-patient. Although this patient ate at Restaurant A, it is possible that this case-patient was infected by a different source and was a sporadic case unrelated to the outbreak. This assumes that the case-patients that were not culture confirmed were infected with Benidorm strain. It is also possible that Denver strain was present in the fountain but was masked by the predominance of Benidorm strain, or that Denver was present in the fountain in early August when this case-patient became ill, but was no longer present in late October when the fountain was sampled. These hypotheses also make it possible that Benidorm strain may have been present in cooling towers but was not present at the time of sampling. If that were the case however, one might have expected more people who had not eaten at Restaurant A to be case-patients. We interviewed approximately half of the controls by phone whereas all case-patients were interviewed in person. This may have led to better recall among cases. Interviews with controls by phone facilitated more rapid recruitment of controls and thus a more timely analysis.

Most of the fountains we sampled had little or no routine maintenance although this is recommended by fountain manufacturers and by the American Society of Heating, Refrigeration, and Air-conditioning Engineers (ASHRAE). ASHRAE guidelines are non-specific for fountain maintenance and are not disseminated widely in the restaurant industry [32]. Proper care and maintenance of ornamental water fixtures, such as decorative fountains, is essential to prevent outbreaks of LD and can be achieved by increasing awareness among fountain operators of the importance of adequate maintenance.

## Conclusion

Small decorative fountains pose a previously unrecognized source of Legionnaires' disease outbreaks as we believe this is the first time that such a small fountain with apparently limited aerosol-generating capability has been implicated as the source of an LD outbreak. Investigations of future community outbreaks of LD should consider exposures to indoor decorative fountains, including small ones such as those that might be present in restaurants, hotels, or other businesses, as potential sources of *Legionella*.

## Competing interests

TK was contracted by SDDH to assist with this outbreak investigation as an environmental engineering consultant specializing in *Legionella *remediation. His consultancy has no conflict of interest with any information contained in this paper.

The author(s) declare that they have no competing interests.

## Authors' contributions

ROL, LK, MCW, CH, TK, BF and MRM participated in the conception and design of the study. ROL, LK, MCW, EB, VS, TK, RB, BF and MRM participated in the analysis and interpretation of data. ROL, MM and LK drafted the article. All authors critically revised the article for important intellectual content and all authors have seen and approved the final version. The corresponding author (ROL) had access to all data and had final responsibility for the decision to submit for publication.

## Disclaimer

The findings and conclusions in this report are those of the authors and do not necessarily represent the view of the Centers for Disease Control and Prevention.

## Pre-publication history

The pre-publication history for this paper can be accessed here:


